# Redefining Facilitation in Nursing Homes: The Critical Role of Frontline Workers in Implementing Care Innovations

**DOI:** 10.1093/geroni/igaf122.693

**Published:** 2025-12-31

**Authors:** Jing Wang, Ruth Anderson, Yinfei Duan, Carole Estabrooks, Anna Beeber

**Affiliations:** University of New Hampshire, Durham, New Hampshire, United States; Duke University, Durham, North Carolina, United States; University of Alberta, Edmonton, Alberta, Canada; University of Alberta, Edmonton, Alberta, Canada; Johns Hopkins University, Baltimore, Maryland, United States

## Abstract

Facilitation in healthcare is traditionally conceptualized as a structured, leadership-driven process within frameworks such as Promoting Action on Research Implementation in Health Services (PARIHS) and integrated-PARIHS (i-PARIHS). However, in nursing homes, where frontline workers—particularly care aides—have the most direct interactions with residents, facilitation occurs informally and is embedded in daily caregiving. This study highlights how frontline workers function as facilitators of care innovations, despite not being formally recognized in existing facilitation frameworks. We conducted qualitative analysis of data from the Translating Research in Elder Care (TREC) program, analyzing 60 in-depth interviews and 150 pages of fieldnotes from three nursing homes. Thematic analysis using ATLAS.ti mapped out the relationships between facilitation, innovation, and implementation strategies, revealing distinct patterns of facilitation. Frontline workers played a key role in bridging gaps between leadership-driven initiatives and the realities of daily care, adapting innovations to fit practical constraints. In settings where facilitation was distributed across multiple staff levels, care aides took initiative in leading pressure ulcer prevention, falls reduction, and dementia behavior management strategies. Conversely, in facilities with hierarchical, top-down facilitation models, staff disengagement and resistance led to ineffective adoption of care innovations. This study challenges traditional paradigms by demonstrating that facilitation in nursing homes is a system-wide, collaborative process rather than solely a leadership function. Recognizing and legitimizing frontline staff as facilitators is critical to sustaining innovation, improving care quality, and fostering adaptive implementation strategies in nursing home settings.

